# Efficacy of Platelet-Rich Plasma Injection in the Management of Rotator Cuff Tendinopathy: A Review of the Current Literature

**DOI:** 10.7759/cureus.26103

**Published:** 2022-06-20

**Authors:** Kavyansh Bhan, Bijayendra Singh

**Affiliations:** 1 Trauma & Orthopaedics, Barts Health NHS Trust, London, GBR; 2 Trauma & Orthopaedics, Medway NHS Foundation Trust, Gillingham, GBR

**Keywords:** leukocyte-rich prp, leukocyte-poor prp, intra-articular prp, platelet-rich plasma therapy for joints, autologous platelet-rich plasma, prp injection, platelet-rich plasma (prp)

## Abstract

Platelet-rich plasma (PRP) is being projected as a newer and superior treatment option for rotator cuff tendinopathy. With the first use of PRP in trauma and orthopedics dating back only to 1998, its advent into the field is relatively recent. Thus, data on long-term efficacy, large cohort studies, or large randomized controlled trials are fairly limited. Moreover, owing to the absence of standardized methods of platelet extraction and lack of consensus on the ideal concentration of platelets in PRP, data comparison from different studies is difficult. Things are complicated by the fact that it is also not clear whether a single injection of PRP is therapeutic or if multiple serial injections are needed to produce the desired effects. The literature on efficacy and pain relief is also obtained from studies with low sample sizes and short follow-ups. The dropout rate and noncompliance rate were also found to be high in some studies. Thus, the data is often not clinically significant and may also be biased due to the non-standardized inclusion and exclusion criteria of the studies. Though multiple studies have suggested good pain control with PRP injection, many studies have found that PRP injection therapy may not be any better than the physical therapy regimen prescribed to patients with rotator cuff tendinopathy. Also, the data on the efficacy of PRP on shoulder function and shoulder range of motion is at variance. This submission aims to evaluate the efficacy and use of PRP in the management of rotator cuff tendinopathy.

## Introduction and background

Shoulder pain is a common and highly disabling complaint, widely regarded as the third most common musculoskeletal complaint globally [1]. Luime et al. estimated that almost 70% of the global population experiences shoulder pain at some point in life [2]. Almost 40%-50% of those complaining of shoulder pain report persistent or recurrent pain at a 12-month follow-up [3]. Rotator cuff tendinopathy is a leading cause of shoulder pain and can be attributed to more than half of the total shoulder pain cases presenting to the orthopedic practice [4]. Its incidence is reported to be higher than 50% in the general population aged above 60 years [5]. Patients diagnosed with rotator cuff tendinopathy suffer from a significant deterioration in their quality of life owing to functional limitation, decreased range of motion, and an inability to perform several actions, which require overhead motions [4].

Rotator cuff tendinopathy can present both acutely as well as chronically. In the acute setting, patients usually present following an injury because of direct trauma to the shoulder [6]. This direct trauma can be a result of obvious injury in contact sports or even poor throwing mechanics in sports such as cricket, baseball, or javelin throw [7]. Chronically, rotator cuff tendinopathy can occur secondary to extrinsic compression, whereby a pathologic contact between the acromion and rotator cuff results in repetitive injuries to the cuff [6]. Alternatively, an acromial spur or a degenerative bursa can also cause mechanical compression, which results in weakened areas of the cuff. These weakened areas can eventually be the cause of full-thickness or partial-thickness tears of the rotator cuff, leading to the development of complaints of pain in the patients.

While success has been seen with treatment modalities like rest, analgesics, physical therapy, and steroid injection for early-stage tendinosis and partial tears, none of these treatment options have shown consistent and reproducible clinical outcomes [8]. It has also been suggested that a failed healing response to microtrauma is one of the biggest barriers to a consistent, reproducible, and effective healing process [8]. The possibility of a decline in stem cell functioning along with deranged angiogenic potential may also contribute heavily to the disrupted healing mechanisms [9]. Platelet-rich plasma (PRP) on the other hand contains various growth factors that are naturally present in the human blood and have the potential to improve the healing response by optimizing the microenvironment for healing [10].

PRP: preparation, contents, and formulation

PRP refers to the processed liquid volume of blood with a platelet concentration above the baseline [11]. While normal platelet count in the blood ranges from 150,000/μl to 350,000/μl, with an average count of about 200,000/μl, a concentration of 1,000,000/μl or above in a 5 ml volume of plasma is what can be considered the defining characteristic of PRP [12]. PRP is invariably derived from autologous blood and is hence inherently compatible and free from risks of transferable diseases like HIV or hepatitis. PRP therapies have long been used for various indications from hair loss to knee osteoarthritis and have generated a lot of interest over the past 30 years [11]. Platelets are microscopic anucleate cell fragments that play a vital role in the maintenance of the coagulation cascade. There are seven known growth factors in PRP; namely, platelet-derived growth factor aa (PDGFaa), PDGFbb, PDGFab, transforming growth factor beta-1 (TGF-b1), TGF-b2, vascular endothelial growth factor (VEGF), and epithelial growth factor (EGF) [12]. The various PRP therapies take advantage of the fact that these growth factors support crucial phases of wound healing and repair cascade, viz inflammation, proliferation, and remodeling.

While platelet concentrations of 1,000,000/μl in autologous plasma may qualify to be termed platelet-rich plasma, current literature lacks any clear consensus on the platelet concentrations and the exact definitions. Hence, we have many papers that define PRP as having platelet concentrations of 1,500,000/μl, while others define PRP as having platelet concentrations of 3,000,000/μl [12]. The PRP can be obtained either by utilizing “in-house techniques” or through the help of readily available commercial kits [13]. PRP is produced by drawing the patient’s own blood, followed by centrifuging a small amount of the withdrawn blood and then extracting the platelet-rich fraction from the centrifuge [14]. The aim is to extract a product that has supra-physiological platelet concentration, which can then be used either in a liquid or an activated gel form. Despite the seemingly similar objectives, various protocols have been developed for PRP preparation. The choice of anticoagulants that are used for blood collection can also affect the final PRP quality. Acid citrate dextrose (ACD) and citrate-theophylline-adenosine-dipyridamole (CTAD) are apparently superior to heparin and citrate in maintaining the integrity of platelet structures, and they help in the prevention of their spontaneous activation [13]. Recent studies have also shown that PRP produced without the use of any additive is more “physiological,” as opposed to PRP produced with the help of additives like ACD [15]. It has a higher platelet yield with a prolonged life of the growth factors.

PRP may be classified as leukocyte-rich PRP (LR-PRP) or leukocyte-poor PRP (LP-PRP). A separation system may be needed to reduce the concentration of leukocytes in PRP. A higher concentration of leukocytes in the PRP can elicit undesired inflammatory responses. It is also argued that while a higher concentration of leukocytes may improve the growth factor yield, the leukocytes can also lead to heightened inflammatory response and disrupt healing by triggering the release of catabolic metabolites [16]. However, there might be a direct relation between high leukocyte and high platelet concentrations [17]. Moreover, the high leukocyte levels also lead to increased antimicrobial activity, which may in turn also improve the availability of growth factors in the PRP [17]. Hence, there is still no clear consensus on the role and efficacy of leukocytes in PRP.

Chahla et al. conducted a systematic review of more than 100 clinical studies and found that only 10% of the studies provided a detailed description of the preparation protocols of PRP, which could be utilized to prepare PRP independently [18]. Most of the studies omit major details like the duration of spin, volume and concentration of leukocytes, platelets in the initial blood samples, or the use of any activating agents along with the final PRP product. This makes it extremely difficult to reproduce the method of PRP preparation safely and consistently. As a result, the market currently has more than 16 commercially available kits for PRP production [18].

PRP is currently one of the most exploited treatment options in clinical practice to provide a regenerative stimulus for tendon healing [19]. Although multiple clinical studies have highlighted the efficacy, safety, and tolerability of PRP in the management of rotator cuff tendinopathy, both in the short term as well as long term, there is no clear consensus on the exact role of PRP in its management. While some studies advocate the use of PRP as a conservative management option, some have recommended its use as a surgical augmentation product. Many studies have also recommended against the use of PRP citing the lack of clear-cut evidence regarding its safety and effectiveness [20]. The purpose and aim of this study are to carry out a comprehensive literature review to be able to contribute to the ongoing clinical and academic debate while attempting to provide clarity for surgeons considering the use of PRP in the management of rotator cuff tendinopathy.

## Review

History of PRP therapy

PRP therapy had its humble origins in the field of hematology. The first use of the word platelet-rich plasma can be dated back to 1954 when Kingsley detailed its applications in patients with severe thrombopenia [21]. The hematologists then used the term "platelet-rich plasma" in the 1970s to describe the plasma which has a platelet count above that of the peripheral blood [22]. It was initially designed to be used as a treatment option for patients suffering from thrombocytopenia [23]. Platelet-rich plasma is synonymous with platelet-rich growth factors, platelet-rich fibrin matrix, and platelet concentrate and is used in the treatment of thrombocytopenia [22]. The extensive use of PRP for platelet transfusion in the treatment of thrombocytopenia resulted in an improvement in the platelet concentrate preparation techniques [24].

In the 1980s, maxillofacial surgeons started using the PRP as platelet-rich fibrin. This fibrin had useful hemostatic and adherence properties and, when coupled with its anti-inflammatory characteristics, had a potential for cell proliferation. The use of PRP in maxillofacial surgery by Marx in the 1990s jumpstarted its use in trauma and orthopedics [25]. Mariani et al. demonstrated the positive effect of PRP in bone healing in maxillofacial surgery in 1998, and this acted as a milestone for the widespread use of PRP in trauma and orthopedics [13]. PRP was widely positioned as a promising regenerative medicine therapy agent. Multiple trials were published, which showed varying results of the efficacy of PRP in conditions like rotator cuff tendinopathy or tendinosis [26]. Randelli et al. were one of the first to discuss the possible use of PRP in conditions like rotator cuff tendinopathy and conducted a successful pilot study demonstrating its efficacy [27]. Subsequently, its use in musculoskeletal medicine, sports injuries, and conditions like lateral epicondylitis and rotator cuff tendinopathy soared [22].

PRP injection in the management of rotator cuff tendinopathy

PRP owing to its theoretical potential to fasten the healing process in various musculoskeletal conditions has attracted a lot of attention in recent days [28]. Although various studies have principally suggested that PRP may have positive clinical effects on conditions like rotator cuff tendinopathy, relatively limited literature supporting its use has been published. Most of the reviews published have been inconclusive with conflicting evidence regarding its efficacy despite the soaring use of PRP in clinical settings worldwide. Most of the data obtained are through studies with small sample sizes and limited follow-up. These studies tend to follow up with PRP patients for short periods and do not have clear data on how the PRP for treatment injections was obtained. The outcome assessments are non-standardized, and the methodology of PRP injection varies from study to study. Through this review, an attempt has been made to present the available data in a systematic, critical, and chronological order to allow the reader to have the best possible chance at evaluating the safety, efficacy, and tolerability of PRP injections in the management of rotator cuff tendinopathy.

Rha et al. in 2013 conducted a single-center, prospective, randomized, and double-blinded study to evaluate the efficacy of PRP injection against dry needling in rotator cuff disease [29]. They recruited a total of 39 patients with either supraspinatus tendinopathy or a partial tear of the supraspinatus tendon for eight months. The dry needling group acted as the control, and both groups were given either a serial dry needling procedure or a serial PRP injection in the affected shoulder at four-week intervals under ultrasound guidance. The patients were prescribed acetaminophen for pain relief during the immediate post-injection period. Details about the concentration of platelets in the PRP were not shared. At the six-month follow-up, the shoulder pain and disability index along with passive shoulder range of motion was utilized to assess the outcomes. It was found that the group which received the PRP injection demonstrated superior clinical relief, as the shoulder pain and disability index was found to be 17.7 ± 3.7 as opposed to 29.5 ± 3.8 in the dry needling group (p < 0.05). The PRP group was also found to be safe, with no adverse clinical effects noted in the group. With regards to the shoulder range of motion, it was found that the patients who received PRP injection had a superior range of motion, with internal rotation and flexion being around four to five times better than the dry needling group. A major limitation of this study was that patients with supraspinatus tear were also included along with the patients who only had rotator cuff tendinopathy. There was no mention of whether these tears were acute or chronic. There was no way to differentiate between the results of these two groups of diverse presentations.

Scarpone et al. published their results of a prospective open-label study evaluating the efficacy of PRP injection in rotator cuff tendinopathy in 2013 [30]. A total of 18 patients were recruited for the study over a period of 56 months, and all had a follow-up until one year. All the patients included had tried a trial of conservative management in the form of physical therapy and corticosteroid injection, which had failed. The patients then received one ultrasound-guided injection of 3.5 mL PRP at the site of the lesion and the surrounding area. The efficacy of the injection was gauged through the visual analog scale (VAS) score for the assessment of pain relief and magnetic resonance imaging (MRI) of the shoulder to assess the severity of tendinopathy through Lewis rubric [31]. At the end of 12 weeks, the patients reported an improvement in the VAS score from 7.5 ± 0.3 to 0.5 ± 0.3. This improved further to 0.4 ± 0.2 (p = .0001) by the end of one year. Sixteen out of the enrolled 18 patients were found to have improved the shoulder MRI severity score at one year. The remaining two patients, although did not demonstrate significant improvement on the MRI, did report improvement in the VAS score. The results from this study demonstrated a significant and sustained improvement in pain for patients suffering from rotator cuff tendinopathy. However, the sample size was small and there was no control group for comparison. Hence, it is difficult to estimate how the patients may have responded over time in the absence of any significant intervention.

Kesikburun et al. in their randomized controlled trial of 40 patients investigated the effects of PRP injection on pain and shoulder function of patients diagnosed with chronic rotator cuff tendinopathy [5]. The diagnosis was made based on the MRI findings of chronic or long-standing rotator cuff tendinopathy along with the presence of a history of shoulder pain for more than three months. Once the diagnosis was confirmed, 40 patients were segregated into a placebo or control group (N = 20) and a PRP group (N = 20). The patients were then injected with either 5 mL of PRP prepared from the venous blood of the patient or 5 mL of saline solution. PRP was obtained using the GPS III Platelet Separation System manufactured by Biomet, Warsaw, Indiana. The platelet count was found to be four times higher than the platelet count in the peripheral blood of the patients. The injections were delivered directly into the subacromial space under ultrasound guidance. The patients were followed up for one year, and the efficacy of the treatment was evaluated through the Western Ontario rotator cuff index (WORC), shoulder pain disability index, VAS score, and an assessment of shoulder range of motion. Although improvement was noticed at all the assessment points of 3, 6, 12, 24 weeks, and one year after injection, a comparison of outcomes revealed no significant difference between the PRP and placebo groups. However, patients of both groups underwent a six-week standard exercise program. Since both the groups underwent this exercise program, it may demonstrate that PRP did not have any additive effect on this exercise regimen. Nevertheless, whether PRP would have had any beneficial effect if there was no exercise program or if improvement may have been seen if serial injections were offered to the patients remains unclear.

Tahririan et al. performed a prospective open-label study of 17 patients enrolled for 26 months to determine the effectiveness of ultrasound-guided PRP injection in patients with chronic rotator cuff tendinopathy [32]. Patients who had failed a trial of conservative management including physiotherapy and had been experiencing symptoms such as shoulder pain or difficulty in overhead movements for more than three months were included. In this study, however, only the patients with less than 1 cm partial tearing of the bursal side of the rotator cuff on MRI studies were included. Patients then received an ultrasound-guided PRP injection through the posterior subacromial approach within 30 minutes to one hour of preparation using the ROOYA GEN® system (Iran) [32]. The platelet count in the PRP was four times the normal platelet count in peripheral blood. The patients were then immobilized for three days with the advice to avoid all types of non-steroidal anti-inflammatory (NSAID) drugs for two days after injections and to take only acetaminophen in situations of pain during these two days. The outcomes were evaluated using the constant shoulder score (CSS) for shoulder function. The evaluation was done once before the injection and once three months after the injection. The CSS includes multiple parameters, namely, pain, activity level, arm positioning, strength of abduction, forward flexion, lateral elevation, external rotation, and internal rotation. It gives a detailed insight into both the functional level as well as the pain level of the shoulder through the scoring. The mean CSS was found to be 37.05 ± 11.03 and 61.76 ± 14.75 before and after the injection respectively (p < 0.001). This suggested that PRP not only provided pain relief but was also helpful in improving the patient’s functional activity. However, it was not clear in the study if the patients were allowed to not take NSAIDs only in the initial post-injection period or if they were not allowed to take them throughout the three-month follow-up period. Moreover, the sample size was small, and the study was an open-label study, making it more prone to bias.

Phadke et al. in their review of the literature published in 2018 suggested that PRP is not only an excellent source of concentrated bioactive molecules but that these biomolecules actually have a strong potential to accelerate the healing of rotator cuff tendinopathies [8]. In their literature review, they concluded that PRP injection was a safe and effective tool in relieving pain and improving function in patients suffering from mild to moderate rotator cuff tendinopathies or early partial rotator cuff tears. However, they noted conflicting evidence when comparing the efficacy of PRP to corticosteroid injections or physical therapy. They were unable to conclude superior treatment options among the three and held unstandardized protocols in the preparation of PRP as one of the major reasons for failure to deduce exact efficacy rates of PRP. Moreover, they noticed that most of the studies reviewed had a very small sample size and none of them explicitly mentioned the return to sporting activities in the younger population. This has serious implications as unless large randomized controlled trials are held upholding the safety, efficacy, and tolerability of autologous PRP injections, widespread adoption of PRP injections will remain difficult. Also, owing to the lack of standardized preparation protocols for PRP and varying concentrations of platelets in PRP, direct comparison of results from different studies is extremely difficult. Phadke et al. also raised the issue of variation in the number of injections given in various studies that were reviewed in their literature review. While most studies advocate a single injection for rotator cuff tendinopathies, many studies have also suggested the use of either serial injections or the use of a second injection in cases where the first injection did not produce the desired results [8]. Hence, owing to the non-standardized protocols, it is difficult to predict the efficacy of PRP injections conclusively.

Kuffler published an interesting case report in 2018 about the differing efficacies of PRP injection for rotator cuff tendinopathy in the same patient at different periods [33]. The patient in the case report was a 67-year-old gentleman with ongoing symptoms in his right shoulder for almost three months. The patient had a traumatic event that probably led to the symptoms three months ago; however, no details regarding diagnosis through an ultrasound scan or MRI scan at the point of injury were available. Regardless, the patient tried conservative measures including NSAIDs and analgesics, but they were found to be ineffective. Upon exhausting all conservative treatment options, he was given the option of a PRP injection. The patient then received a PRP injection and experienced almost 98% improvement in pain at the three-month follow-up. The patient also showed significant physical improvement, with him being able to carry out all his daily activities virtually pain-free. Unfortunately, the patient developed similar symptoms on the contralateral shoulder seven months later. The patient had an ultrasound scan that confirmed a diagnosis of rotator cuff tendinopathy. He initially tried a slew of conservative measures like NSAIDs and analgesics but was not relieved of symptoms. The same physician then injected PRP into the contralateral shoulder under similar conditions and approach. However, in this instance, the patient did not show any improvement and was still in considerable pain at the six-week follow-up. The author has stated that the concentration, quantity, and approach remained the same for both the injections. The patient then received a corticosteroid injection and experienced immediate improvement in his pain symptoms. This case study had an added benefit of a long follow-up. The shoulder that was injected with PRP was followed up for 26 months, and the patient was consistent until the last follow-up. However, this case report did not give any details on how the PRP was obtained and which system was used in its preparation. It also did not give any details on the exact concentration of PRP, whether it was a leukocyte-rich or leukocyte-poor PRP. Details regarding the exact technique of injection and the exact quantity were also missing. Owing to the lack of information, strong inferences may not be drawn from this study. The authors have suggested that the difference in results may be due to the patient’s different physiological status at the time of obtaining the blood samples. However, such claims would need further studies to be relied upon.

In their prospective randomized controlled trial of 64 patients, Lee et al. compared the clinical outcomes of exercise treatment and PRP injection therapy in patients diagnosed with rotator cuff tendinopathy [34]. The injections were given as an outpatient-based treatment to patients who were diagnosed with rotator cuff tendinopathy with no rotator cuff tears and had failed to respond to other conservative measures for more than one month. The patients who were given PRP injections were given the injections under ultrasound guidance in the outpatient setup. The PRP injection group had been further subdivided into leukocyte-rich PRP and leukocyte-poor PRP. A total of 1.5 mL of either leukocyte-rich PRP or leukocyte-poor PRP was injected into the supraspinatus. The PRP was obtained through the ACMTM kit, with leukocyte-poor PRP having a platelet concentration of three times the peripheral count and leukocyte-rich PRP having a platelet count of six times the peripheral count. The injections were delivered into the degenerative area of the supraspinatus muscle, while the patient was seated in an upright position with maximum external rotation of the shoulder. The patients were then followed up for six months, and the outcome was assessed through the numeric rating scale (NRS) for evaluation of pain, American Shoulder and Elbow Society (ASES) score, and CSS for evaluation of shoulder function at baseline, three months, and six months. While the NRS involves a patient’s self-assessment of the intensity of pain on a scale of 1 to 10, the ASES score comprises the assessment of both pain and activities of daily living. The outcome assessment showed an improvement in ASES score from 60.21 to 66.12 at three months in the exercise treatment group, while it showed a much larger improvement to 73.89 at three months for the PRP injection group from a baseline of 57.93. The scores further improved to 75.88 and 80.3, respectively, at the six-month follow-up. Although a statistically significant difference was seen between the exercise treatment and PRP groups, no obvious difference in the outcomes could be noted between the leukocyte-rich PRP and leukocyte-poor PRP groups. This may suggest that the platelet concentration higher than three times the peripheral blood may have a limited role in improving the efficacy of PRP injection treatment. Moreover, this study did not have an injection of an anesthetic agent prior to the actual PRP injection, which may suggest that the results from this study are more reliable as compared to many other studies that involve an injection of an anesthetic agent into the shoulder prior to the PRP injection.

Flatow recently published the results of their double-blind randomized controlled trial comparing the effectiveness of PRP with corticosteroid injections in controlling pain and improving shoulder function in patients diagnosed with rotator cuff tendinopathy [35]. The study had a relatively large sample size of 99 patients. The patients had their diagnosis of tendinopathy confirmed through an ultrasound scan or an MRI scan. Only those patients who had the symptoms of rotator cuff tendinopathy for at least three months and had tried conservative measures like physical therapy or corticosteroid injections but failed were included. Patients who had corticosteroid injections in the month prior to the enrolment date were not included. Once enrolled in the study, the patients received an injection of either a corticosteroid or PRP under ultrasound guidance. In the group receiving PRP injection, leukocyte-poor PRP prepared through RegenLab prepackaged kit (Regen Lab, Lausanne, Switzerland) was injected with 3-5 mL of PRP directly into the site of pathology in the supraspinatus tendon, with residual PRP (PRP unused after injecting from a total of 5.5 mL PRP produced) being injected into the subacromial space. The patients in the corticosteroid group had infiltration of triamcinolone into the subacromial bursa instead of the tendon. The outcome analysis was primarily done through a VAS scoring at six weeks, three months, and 12 months. Functional assessment through the ASES scoring was also done at the same time points. The assessment showed that although both corticosteroid injections and PRP injections were helpful in alleviating the pain and symptoms of tendinopathy, PRP was not necessarily superior to corticosteroid injection. It was found that the improvement in VAS score was comparable in both the intervention methods at all follow-up intervals except the three-month follow-up, wherein PRP was slightly superior to corticosteroid. Similar results were also noticed on the ASES scoring. Although both the intervention methods showed an improvement in overall shoulder function levels, PRP was not statistically superior to corticosteroid at the 12-month follow-up. A point of note is that this study utilized a leukocyte-poor PRP for intervention, whereas multiple studies have postulated that a higher concentration of leukocytes is more conducive to healing in tendinopathies [36]. Another limitation of the study was that the authors excluded patients “who had a high risk of associated litigation” but failed to define how these patients were identified, thus leaving the study prone to bias. Moreover, there was no control group in the study as both the groups received some form of intervention; hence, individual efficacy of PRP and corticosteroid injections cannot be deduced from the study. A summary of the papers reviewed has been presented in Table 1.

**Table 1:** Summary of the papers reviewed LOE: Level of evidence; Cont: Control; Int: Intervention.

Study	LOE	Study Type	Patients (Cont/Int)	Control v/s Intervention	Results	Critique
Rha et al. [29]	Level II	RCT	(20/19)	Dry needling v/s PRP	PRP: Superior pain relief, superior arm function	High follow-up loss: 25% small sample size
Scarpone et al. [30]	Level IV	Cohort study	18	-	Significant pain relief, improvement in shoulder range of motion, improvement in rotator cuff integrity on MRI	Small sample size, absence of control
Kesikburun et al. [5]	Level II	RCT	(20/20)	Exercise regimen v/s PRP	Similar results in both groups	Small sample size, selection bias by including only those patients who showed 50% improvement in pain after injection of anesthetic agent
Tahririan et al. [32]	Level IV	Cohort study	17	-	Significant improvement in pain and range of motion	Small sample size, absence of control, short follow-up of three months
Phadke et al. [8]	Level I	Literature review	-	-	PRP is safe and effective in improving pain and shoulder function, but no recommendation upon comparison with steroids	Descriptive literature review
Kuffler [33]	Level VI	Case Report	1	-	Improvement in shoulder pain and range of motion in one shoulder, no effect on the other shoulder of the same patient	Single case report, may be an isolated case
Lee et al. [34]	Level II	RCT	(34/30)	Exercise regimen alone v/s PRP + Exercise regimen	Superior improvement in shoulder pain and range of motion in the PRP + Exercise regimen group as compared to the standalone exercise regimen group	Small sample size, short follow-up, PRP group also received exercise regimen, which may mask the actual effects of PRP
Flatow [35]	Level II	RCT	(52/47)	Corticosteroid v/s PRP	Superior improvement in pain and shoulder range of motion at three months for PRP, no difference in improvement at 12 months	Difference in baseline outcome scores of both groups with PRP group starting with a worse-off sample size
Lin et al. [10]	Level I	Meta-analysis	-	-	PRP effective in providing pain relief in rotator cuff tendinopathy; no positive effect on improving shoulder function	Heterogeneity in diagnostic criteria among the reviewed trials

Discussion

PRP is now being increasingly used to treat rotator cuff tendinopathy, although corticosteroid injections and physiotherapy remain the mainstay of conservative management [35]. The goal of any conservative treatment regime is a reduction in pain and improvement in the shoulder function or shoulder range of motion. Having been used widely as a treatment option for various shoulder pathologies, interest has grown manifold in the role of PRP in conditions like rotator cuff tendinopathy and elbow epicondylitis [37]. Randomized controlled trials, retrospective cohort studies, case series, and case reports have all demonstrated a possible role of PRP in rotator cuff tendinopathy [38]. However, the results are not unequivocal.

Although data from multiple studies have suggested that PRP is a cost-effective and safe treatment option with low associated morbidity, there is still no consensus in the literature about its widespread use [8]. This may be attributed to the paucity of data along with multiple low-quality studies possibly affecting the results. Despite various studies suggesting statistically significant differences in the outcomes of PRP injection when compared to controls, or corticosteroid injections, almost none of them describe the minimal clinically important difference. It is now widely known that although studies may find statistically significant differences in the outcomes, this does not necessarily translate to a noteworthy difference in the clinical outcomes [39]. Moreover, with highly variable methods of PRP preparation along with different definitions of what constitutes PRP, it is extremely difficult to compare the outcomes of different studies. Also, multiple studies also had different types of PRP being used, namely, leukocyte-rich PRP and leukocyte-poor PRP, which may also act differently on the rotator cuff pathologies, thereby producing varying results. This is also complicated by the fact that various studies suggest serial injections of PRP rather than a single-dose injection.

PRP does seem to play an effective role in significantly reducing the retear rate in acute rotator cuff tears and in preventing full-blown large tears in chronic rotator cuff tendinopathies [39]. Since the retear rates after an arthroscopic repair of rotator cuff tear continue to be quite high, biologic methods of managing rotator cuff pathologies like PRP are gaining traction since they are said to improve bone-tendon healing, thereby reducing the chances of rupture, tear, or retears [40]. A rotator cuff tendinopathy is characterized by the presence of numerous microtears that possess the ability to transform into a full-blown tear. The PRP may reduce the chances of the progression of microtears into full tears and also reduce the chances of a retear as it has been found that patients who receive a PRP injection in their cuff tend to have a lower infiltration of fatty cells into the rotator cuff, thereby strengthening the rotator cuff and reducing the possibility of a tear. However, prevention of retear is an extremely difficult metric to compare since there are vast differences among the studies including the heterogenicity of the populations and absence of any minimal clinically important difference criteria as all retears are labeled as a clinically significant finding [39]. Miranda et al. in their systematic review of current clinical evidence supporting the use of PRP in rotator cuff pathologies suggested that although most of the preclinical or laboratory studies report encouraging and positive results for PRP in rotator cuff pathologies, the same success is not translated into clinical settings with almost 70.6% clinical studies suggesting minimal to no difference between PRP group and control group [41]. However, various other studies have suggested both a short-term as well as a long-term pain reduction and functional improvement with a single PRP injection into the rotator cuff [29].

Through this review, it has been ascertained that PRP injection, corticosteroid injection, and physiotherapy or physical therapy are the best and most commonly used options available to clinicians for the conservative management of chronic rotator cuff tendinopathies. Out of the available options, PRP and physical therapy can be assumed to be the safest as there are no significant adverse events reported with the use of either in the current evidence base. Multiple studies, however, have shown the varying incidences of adverse events with corticosteroid injections. Adverse events reported range from headache, arterial hypertension, facial erythema to facies lunata, and full-blown anaphylaxis [42]. Despite most of the studies having reported mild to moderate adverse events with corticosteroids, the possibility of serious events like anaphylaxis is always present with the use of corticosteroids. As PRP used for rotator cuff pathologies is autologous in nature, the chances of a serious adverse event like anaphylaxis are quite low, making it an effective and safe intervention in comparison to corticosteroids.

## Conclusions

The extensive literature found on this topic was aimed at finding an alternative and effective treatment option for rotator cuff tendinopathy. Although multiple treatment options like corticosteroid injections, dry needling, and targeted exercise regimens have been tried, their low compliance and efficacy along with chances of adverse effects have forced the physicians to try alternative methods like PRP injection. It can be agreed that PRP injection offers an efficient alternative for improving the shoulder function and range of motion in patients suffering from rotator cuff tendinopathy. In patients with restricted shoulder movements, the improvements in DASH, ASES, or WORC scores have shown that PRP as a standalone treatment option can help improve the shoulder range of motion. However, the absence of any long-term follow-up data means that no comments about refractory rotator cuff tendinopathy after a PRP injection can be made. Moreover, since rotator cuff tendinopathy is a chronic condition, short-term improvement in the range of motion may not imply that the condition will not worsen over time. Nonetheless, PRP may be safely suggested to improve the shoulder range of motion in periods up to at least one year after injection in the majority of patients without major adverse effects. More definitive studies with standardized methods of preparations and follow-ups are required to make a conclusive recommendation.
